# Supplementary material for: Machine learning for predicting cognitive deficits using auditory and demographic factors
Source: PLoS One. 2024 May 14;19(5):e0302902. doi: 10.1371/journal.pone.0302902 (PMC11093307; doi:10.1371/journal.pone.0302902)
Supplement: S1 File — (DOCX) [file pone.0302902.s001.docx]

**Supplementary**

**1)**

*Supplementary Figure 1: Summary of Subject Selection Process for Machine Learning Analysis*

**2)**

education_years

meanSNR

PTA_R

PTA_L

SRT_composite

age

score

gap_detection_threshold_both

scoreslope

SRT_compositeslope

CD4_countslope

PTA_Rslope

gap_detection_thres_bothslope

CD4_count

PTA_Lslope

meanSNRslope

A)

B)


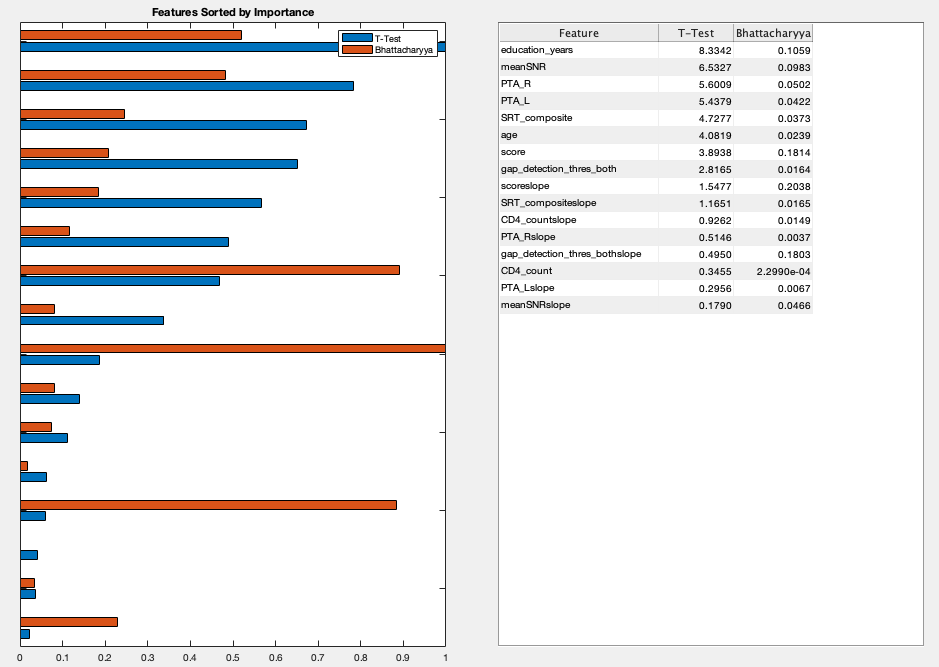


Supplementary Figure 2: Diagnostic feature ranking results which show the predictive capability rankings of prospective features using a T-Test ranking algorithm and a Bhattacharyya ranking algorithm for the binary classification variable derived from the MoCA score (<26 = impaired) A) Bar graph representation with normalized scores B) numerical representation with absolute scores

**3)**

Supplementary Table 1: Hyperparameter sweep for Kernel Distribution (No hyperparameters for Gaussian Naïve Bayes)

| **Hyperparameter Search Range** | |
| --- | --- |
| *Distribution Name* | *Kernel Type* |
| Kernel | Gaussian, Box, Epanechnikov, Triangle |
| **Suggested Optimized Hyperparameters** | |
| *Distribution Name* | *Kernel Type* |
| Kernel | Gaussian |

**4)**

**Background on Naïve Bayes Algorithms:**

The algorithm that we found to provide the most accurate results were two naïve Bayes algorithms, Gaussian Naïve Bayes and Kernel Naïve Bayes. For these algorithms, the ML uses a paradigm constructed using statistical models based on Bayes’ Theorem [24]. Generally, the goal of Bayesian ML is to estimate the posterior distribution (𝑝(𝜃|𝑥)p(θ|x)) given the likelihood (𝑝(𝑥|𝜃)p(x|θ)) and the prior distribution, 𝑝(𝜃)p(θ). The posterior probability is the revised or updated probability of an event occurring after taking into consideration new information. The likelihood is something that can be estimated from the training data that is provided to the ML model. It then performs a *Maximum a Posteriori (MAP)*, an iterative process which updates the model’s parameters to maximize the posterior distribution which takes the training data as fixed and determines the probability of any parameter setting 𝜃 given that data. So, from a high level, through conditional probabilistic computation Naïve Bayes classification algorithms aim to optimally map new data into classification sets [24]. For this study the Naïve Bayes methodology outperformed other algorithms which could be a consequence of the characteristics of the features.
